# Application of machine learning models in predicting insomnia severity: an integrative approach with constitution of traditional Chinese medicine

**DOI:** 10.3389/fmed.2023.1292761

**Published:** 2023-10-19

**Authors:** Shenguang Li, Po Zhu, Guoying Cai, Jing Li, Tao Huang, Wenchao Tang

**Affiliations:** ^1^Shanghai Minhang Hospital of Integrated Traditional Chinese and Western Medicine, Shanghai, China; ^2^Yueyang Hospital of Integrated Traditional Chinese and Western Medicine Affiliated to Shanghai University of Traditional Chinese Medicine, Shanghai, China; ^3^School of Acupuncture-Moxibustion and Tuina, Shanhgai University of Traditional Chinese Medicine, Shanghai, China

**Keywords:** machine learning, insomnia, constitution of traditional Chinese medicine, prediction model, random forest classifier (RFC), support vector classifier (SVC), K-nearest neighbors (KNN)

## Abstract

**Objective:**

This study sought to explore the utility of machine learning models in predicting insomnia severity based on Traditional Chinese Medicine (TCM) constitution classifications, with an aim to discuss the potential applications of such models in the treatment and prevention of insomnia.

**Methods:**

We analyzed a dataset of 165 insomnia patients from the Shanghai Minhang District Integrated Traditional Chinese and Western Medicine Hospital. TCM constitution was assessed using a standardized Constitution in Chinese Medicine (CCM) scale. Sleep quality, or insomnia severity, was evaluated using the Spiegel Sleep Questionnaire (SSQ). Machine learning models, including Random Forest Classifier (RFC), Support Vector Classifier (SVC), and K-Nearest Neighbors (KNN), were utilized. These models were optimized using Grid Search algorithm and were trained and tested on stratified patient data, with the TCM constitution classifications serving as primary predictors.

**Results:**

The RFC outperformed others, achieving a weighted average accuracy, precision, recall, and F1-score of 0.91, 0.94, 0.92, and 0.92 respectively, it also effectively classified the severity of insomnia with high area under receiver operating characteristic curve (AUC-ROC) values. Feature importance analysis demonstrated the Damp-heat constitution as the most influential predictor, followed by Yang-deficiency, Qi-depression, Qi-deficiency, and Blood-stasis constitutions.

**Conclusion:**

The results demonstrate the potent utility of machine learning, specifically RFC, coupled with TCM constitution classifications in predicting insomnia severity. Notably, the constitution classifications such as Damp-heat and Yang-deficiency emerged as crucial determinants, emphasizing its potential in guiding targeted insomnia treatments. This approach enables the development of more personalized and efficient interventions, thereby enhancing patient outcomes.

## 1. Introduction

Insomnia, a common sleep disorder, disrupts the ability to fall asleep, maintain sleep, or achieve restorative sleep, consequently interfering with daytime functioning (1). It is a significant public health issue, affecting approximately 10–30% of the global population and causing further health complications (2). This concern is amplified in elderly and psychiatric demographics, where its prevalence is markedly higher (3). Given the complex etiology of insomnia, which often encompasses an intricate interplay of biological, psychological, and environmental factors, crafting effective, individualized treatment strategies remains a considerable challenge for both primary care providers and sleep medicine specialists (4–6).

Traditional Chinese Medicine (TCM), with its unique health and disease perspective that emphasizes harmony among body, mind, and environment, has been suggested as a complementary approach to the conventional biomedical model for managing insomnia (7, 8). Within this framework, individual inherent traits, or “constitution,” are central. These constitutions encompass physical characteristics, susceptibility to diseases, and reactions to environmental changes and are assessed using the standardized Constitution in Chinese Medicine (CCM) scale. This scale, with validated reliability, is used in various health contexts, forming a basis for understanding individual differences in health and disease from a TCM perspective (9–12). Building on this foundational concept of constitution in TCM, insomnia is interpreted as more than just a symptom; it is a manifestation of the imbalance within the body’s fundamental elements such as Yin and Yang. This perspective aligns with the holistic nature of TCM, which perceives sleep disturbances as interconnected with other physiological and psychological imbalances. Historically, personalized therapeutic strategies based on individual constitution and presenting symptoms were formulated by TCM practitioners. These strategies aimed at restoring balance and harmony within the body, addressing the root causes of insomnia rather than merely alleviating the symptoms. Therefore, the ancient practices and holistic approach of TCM provide a comprehensive viewpoint to explore the underlying intricacies of insomnia and its relationship with various constitutions (13, 14).

Advancing from this holistic viewpoint and the significant role of constitution in TCM, recent empirical studies suggest the potential applicability of the CCM in managing insomnia. Specific TCM constitution types, such as Yin-deficiency and Qi-deficiency, have been recognized as more prevalent in individuals grappling with insomnia (15). Further, a study by He et al. (16) reported that acupuncture treatment based on an individual’s CCM score led to notable improvements in sleep quality and a reduction in insomnia symptoms. In the face of the widespread prevalence and complex nature of insomnia, these findings indicate a promising avenue for incorporating the TCM constitution-based approach into a comprehensive, individualized management plan for this sleep disorder (14).

This study underscores the significant influence of TCM constitution on the prevention and prognosis of insomnia, suggesting that the CCM scale score could act as a crucial predictive tool for determining the severity of insomnia. The uniquely powerful role of an individual’s constitution in TCM highlights its impact on overall physical and mental health, with a marked effect on susceptibility and severity of illnesses, including insomnia (17). By exploring the predictive capabilities of the CCM scale in relation to insomnia severity, we are not only poised to forge innovative links between TCM and modern sleep medicine, but also lay the foundation for the development of more comprehensive, personalized therapeutic strategies that emphasize the remarkable contributions of TCM constitution in managing and predicting insomnia, thus shaping its treatment outcomes.

Simultaneously, the advent of machine learning algorithms in healthcare presents a transformative opportunity to unearth complex relationships between variables (18). Particularly, methods like Random Forest (19), Support Vector Machine (20), and K-nearest neighbors (21) have been extensively utilized in the field of predictive medicine and have shown robust results in a variety of clinical prediction tasks (22, 23). Incorporating these algorithms to analyze the CCM scale scores might offer novel insights into the multifaceted association between TCM constitution types and insomnia severity.

While the ancient wisdom of TCM has a longstanding history in addressing insomnia, encapsulating holistic and individualized approaches, there remains a striking scarcity in modern studies with rigorous data support directly linking TCM constitution types with insomnia severity. This discernible gap in evidence-based research underscores the need for more in-depth investigations that explore the predictive role of TCM constitution in insomnia, integrating machine learning methodologies. Such exploration can deepen our understanding of insomnia through a TCM lens and contribute valuable insights towards the pathogenesis, prediction, and treatment of insomnia (24).

To this end, our study aims to examine the correlation between the CCM scale score and the severity of insomnia, utilizing machine learning algorithms for prediction. We anticipate that our findings will establish a theoretical and empirical groundwork for the application of TCM constitution in insomnia prediction and management. This, in turn, would foster a more tailored and efficacious approach to insomnia treatment, potentially improving patient outcomes and quality of life.

## 2. Methods

### 2.1. Data sources

This investigation was conducted with the explicit approval from the Ethics Committee of the Shanghai Minhang District Integrated Traditional Chinese and Western Medicine Hospital (Ethics Reference No. 2021–007) and each included patient provided a written informed consent. The data utilized in this study were meticulously collected from 165 patients diagnosed with insomnia, receiving their treatment in the Department of Preventive Medicine of the aforementioned hospital during the period of November 2021 to December 2022. The sample included 110 females with an average age of 46.92 ± 12.38 years, and 55 males with an average age of 46.05 ± 13.01 years.

#### 2.1.1. Inclusion criteria

The inclusion criteria were established in accordance with the diagnostic criteria for primary insomnia in the 3rd Edition of the Chinese Classification and Diagnostic Criteria of Mental Disorders (CCMD-3), and the diagnostic criteria for insomnia in the “Diagnostic Criteria and Therapeutic Effect of TCM Diseases and Syndromes.” The criteria were as follows: (1) Age between 18 and 75 years; (2) Sleep disorder is the primary symptom, with other symptoms secondary to insomnia. The main symptoms include difficulty in falling asleep, shallow sleep, excessive dreaming, early waking, and difficulty falling back asleep after waking. Secondary symptoms include palpitations, forgetfulness, dizziness, fatigue, a sallow complexion, among others. All of the main symptoms and at least one of the secondary symptoms should be present; (3) The sleep disorder occurs at least three times a week and lasts for more than a month; (4) Insomnia causes significant distress or some symptoms of mental disorder, leads to decreased efficiency in activities or hampers social functioning; (5) Insomnia is not due to any physical disease or mental disorder.

#### 2.1.2. Exclusion criteria

Subjects were excluded from the study if they: (1) did not meet the aforementioned inclusion criteria; (2) were pregnant or breastfeeding women; (3) had used antipsychotics or antidepressants within a week before their consultation; (4) had serious organ dysfunction or severe diseases in other systems; (5) had serious mental disorders; (6) were patients with malignant tumors; (7) had drug dependency.

### 2.2. Observational indicators

#### 2.2.1. TCM constitution evaluation

TCM constitution was assessed using the CCM scale. This scale is composed of nine TCM constitution classifications, specifically Balanced constitution, Qi-deficiency, Yang-deficiency, Yin-deficiency, Phlegm-dampness, Damp-heat, Blood-stasis, Qi-depression, and Special constitution, each comprising 6–8 items. The characteristics of these constitution classifications are listed in Table 1.

**Table 1:** The characteristics of nine TCM constitution classifications.

TCM constitution classification	Characteristic
Balanced constitution	Individuals have a strong physique, stable emotions, and good adaptability. They rarely get sick and recover quickly.
Qi-deficiency	Individuals tend to have weak muscles, low energy, and poor immunity. They are easily fatigued, catch colds frequently, and sweat spontaneously.
Yang-deficiency	Individuals tend to have cold limbs, low metabolism, and slow pulse. They are sensitive to cold and dampness, and prefer warm foods and drinks.
Yin-deficiency	Individuals tend to have dry skin, hair, and mouth, hot sensations in the palms and soles, and night sweats. They are prone to heat-related diseases and insomnia.
Phlegm-dampness	Individuals tend to have overweight body, oily skin, and sticky tongue coating. They often feel heavy, sluggish, and bloated. They are susceptible to metabolic disorders and chronic diseases.
Damp-heat	Individuals tend to have yellowish complexion, red eyes, and bitter taste in the mouth. They often suffer from inflammation, infection, and skin problems. They are intolerant of hot and humid weather.
Blood-stasis	Individuals tend to have dark or purple complexion, lips, and nails, pain or numbness in certain areas, and irregular menstruation or bleeding. They often have poor blood circulation and clotting problems.
Qi-depression	Individuals tend to have emotional fluctuations, chest tightness, and sighing. They often experience stress, frustration, and depression. They are vulnerable to digestive and mental disorders.
Special constitution	Individuals have some congenital or genetic abnormalities that affect their health or appearance. They may have allergies, deformities, or rare diseases.

Each item provides five potential responses, ranging from “none” to “always,” scored from 1 to 5, respectively. The original score is obtained by summing up the scores of each item. Subsequently, the converted score is calculated using the formula: (original score - number of items) * 100 / (number of items * 4). A converted score of ≥60, provided that the converted scores of the other eight biased constitutions are all <30, is deemed a definitive (“yes”) constitution classification. A score < 40 is considered indicative (“basically yes”) of a classification, while all other cases are determined as negative (“no”) for the specific classification.

For this study, rather than establishing a constitution determination, we opted to employ the converted scores across all categories, given that these scores reflect the comprehensive constitutional characteristics of the patients. Hence, the converted scores of the nine classifications were input as independent variables (X) into the machine learning model for predicting insomnia severity.

#### 2.2.2. Sleep quality evaluation

Sleep quality in this study was evaluated using the Spiegel Sleep Questionnaire (SSQ), an established and validated (Cronbach’s α coefficient of SSQ is 0.868) self-reported instrument routinely employed in clinical research to assess sleep–wake patterns. This tool boasts comprehensive coverage of the sleep–wake cycle, contributing to its sensitivity and reliability in tracking sleep pattern changes over time, as well as evaluating the efficacy of sleep-related interventions. It remains particularly invaluable in the study and management of sleep disorders, including insomnia (25, 26).

The SSQ explores six dimensions of sleep and wakefulness: initial and terminal insomnia, perceived quality of sleep, refreshment upon awakening, daytime alertness, and total sleep time. Each dimension is assessed on a 5-point Likert scale, with higher scores corresponding to worsened sleep disturbances. Thus, a lower cumulative score signifies improved sleep quality and decreased daytime sleepiness.

For the purposes of this investigation, insomnia severity was delineated into three classes, according to the SSQ scores of the included cases: mild (score ≥ 12), moderate (score ≥ 18), and severe (score ≥ 24). The outcomes of these three severity classifications will serve as the dependent variable (Y) in the training of our machine learning model.

### 2.3. Data analysis

The predictive power of TCM constitution classifications on the severity of insomnia was explored using three machine learning models: Random Forest Classifier (RFC), Support Vector Classifier (SVC), and K-Nearest Neighbors Classifier (KNN). The data set was partitioned into a training set (80% of the total data) and a test set (20% of the total data), enabling model training and subsequent performance evaluation.

#### 2.3.1. Data preprocessing

Data preprocessing involved normalization to maintain uniformity in feature scales, imputation of missing values through domain-specific insights and statistical methods, and the handling of outliers to reduce skewness and model bias, ensuring the reliability and validity of the dataset.

#### 2.3.2. Feature selection

The feature selection was driven by the TCM constitution assessment results acquired through the CCM scale. The converted scores derived from the nine TCM constitution classifications served as pivotal features in our models.

#### 2.3.3. Model optimization and hyperparameter tuning

In our relentless pursuit of model optimization, a Grid Search algorithm was meticulously implemented to fine-tune the hyperparameters of each model, assessing a range of parameter values to identify the optimal combination enhancing model performance. For the RFC, the parameters under consideration included ‘n_estimators’ (the number of trees in the forest), which was varied among [10, 50, 100, 200, 500], ‘max_depth’ (the maximum depth of the tree), taking values from [None, 10, 20, 30, 50], and ‘min_samples_split’ (the minimum number of samples required to split an internal node), taking values from (2, 5, 10).

For the SVC, the parameters ‘kernel’ type, varied among [‘rbf’, ‘linear’, ‘poly’], ‘C’ (the penalty parameter), ranging from [0.1, 1, 10, 100, 1,000], and ‘gamma’ (a parameter affecting the shape of the decision boundary), with values from [‘scale’, ‘auto’] were tuned. Finally, for the KNN model, ‘n_neighbors’ (the number of neighbors to include in the majority of the voting process), varied among (3, 5, 10, 20), and ‘weights’ (the weight function used in prediction), taking values from [‘uniform’, ‘distance’], were adjusted.

#### 2.3.4. Model performance evaluation

Model performance was evaluated using several key indicators, including accuracy, precision, recall, f1-score and area under the receiver operating characteristic curve (AUC-ROC).

Accuracy (A) is the proportion of true results (both true positives and true negatives) among the total number of cases examined, and calculated as:


(1) $$A=\frac{\left(TP+TN\right)}{\left(TP+FP+TN+FN\right)}$$

where TP is the number of true positives, FP the number of false positives, TN is the number of true negatives, and FN is the number of false negatives.

Precision (P) quantifies the number of positive class predictions that actually belong to the positive class, and is defined as:


(2) $$P=\frac{TP}{\left(TP+FP\right)}$$

Recall (R) quantifies the ability of a model to find all the relevant cases within a dataset, and is defined as:


(3) $$R=\frac{TP}{\left(TP+FN\right)}$$

The F1-score (F1) is the harmonic mean of precision and recall, calculated as:


(4) $$F1=\frac{2PR}{\left(P+R\right)}$$

AUC-ROC is a performance measurement for the classification problems at various threshold settings, representing the degree or measure of separability and indicating how well the model can distinguish between classes. In this work, AUC-ROC was used in evaluating the predictive accuracy of machine learning models in distinguishing between different severity levels of insomnia. Each model’s performance was evaluated on the test set to ensure the evaluation is unbiased and reflects the model’s ability to generalize to unseen data.

**Figure 1:**
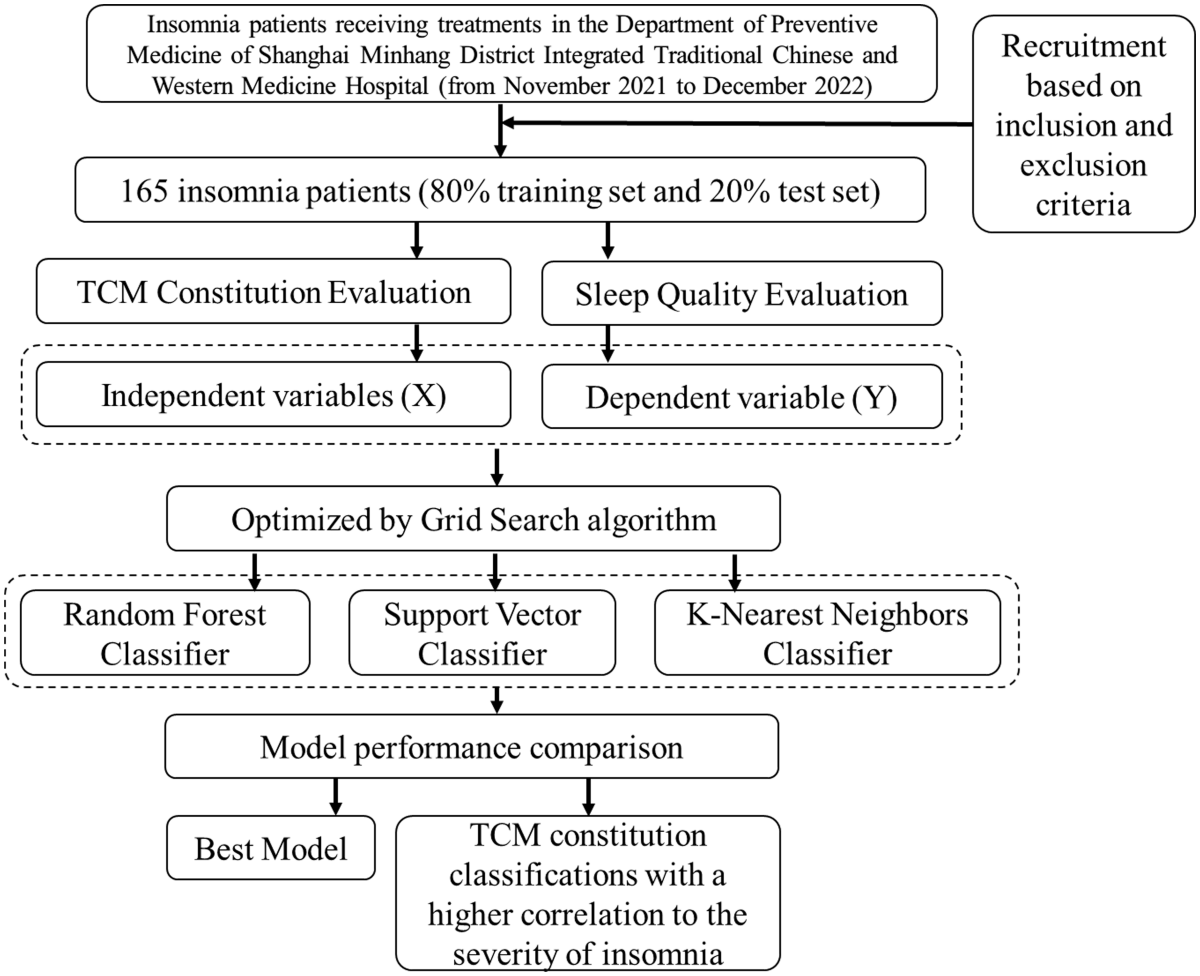
Overview of the data acquisition, modeling, and model performance evaluation.

In addition to building the predictive models, correlation analyses were performed to assess the relevance of the nine TCM constitution classifications to insomnia severity. Those constitutions demonstrating a higher correlation were considered as key features, enriching the predictive models and enhancing their practical applicability in insomnia management. The whole research workflow is shown in Figure 1.

## 3. Results

The Basic information, TCM constitution converted scores and SSQ scores of insomnia patients were listed in Table 2. Three machine learning models – RFC, SVC, and KNN - were utilized to discern patterns in TCM constitution converted scores, with the aim to predict insomnia severity.

**Table 2:** Basic information and evaluation results of TCM constitution and sleep quality of insomnia patients.

Indicators	Female	Male
Number	110	55
Age (Year)	46.92 ± 12.38	46.05 ± 13.01
Course of disease (Year)	2.5 ± 1.72	2.37 ± 1.64
Balanced constitution converted score	32.57 ± 9.12	35.71 ± 12.64
Qi-deficiency converted score	37.29 ± 10.91	32.81 ± 13.8
Yang-deficiency converted score	29.57 ± 14.36	20.67 ± 16.79
Yin-deficiency converted score	32.58 ± 13.09	31.82 ± 11.08
Phlegm-dampness converted score	21.37 ± 10.38	18.42 ± 10.05
Damp-heat converted score	18.14 ± 11.14	16.16 ± 10.09
Blood-stasis converted score	35 ± 10.61	29.41 ± 11.77
Qi-depression converted score	31.8 ± 13.35	26.5 ± 13.1
Special constitution converted score	10.42 ± 13.18	5.26 ± 6.98
SSQ score	22.16 ± 6.05	22.09 ± 6.3
Number of patients with mild insomnia	33	15
Number of patients with moderate insomnia	37	18
Number of patients with severe insomnia	40	22

**Figure 2:**
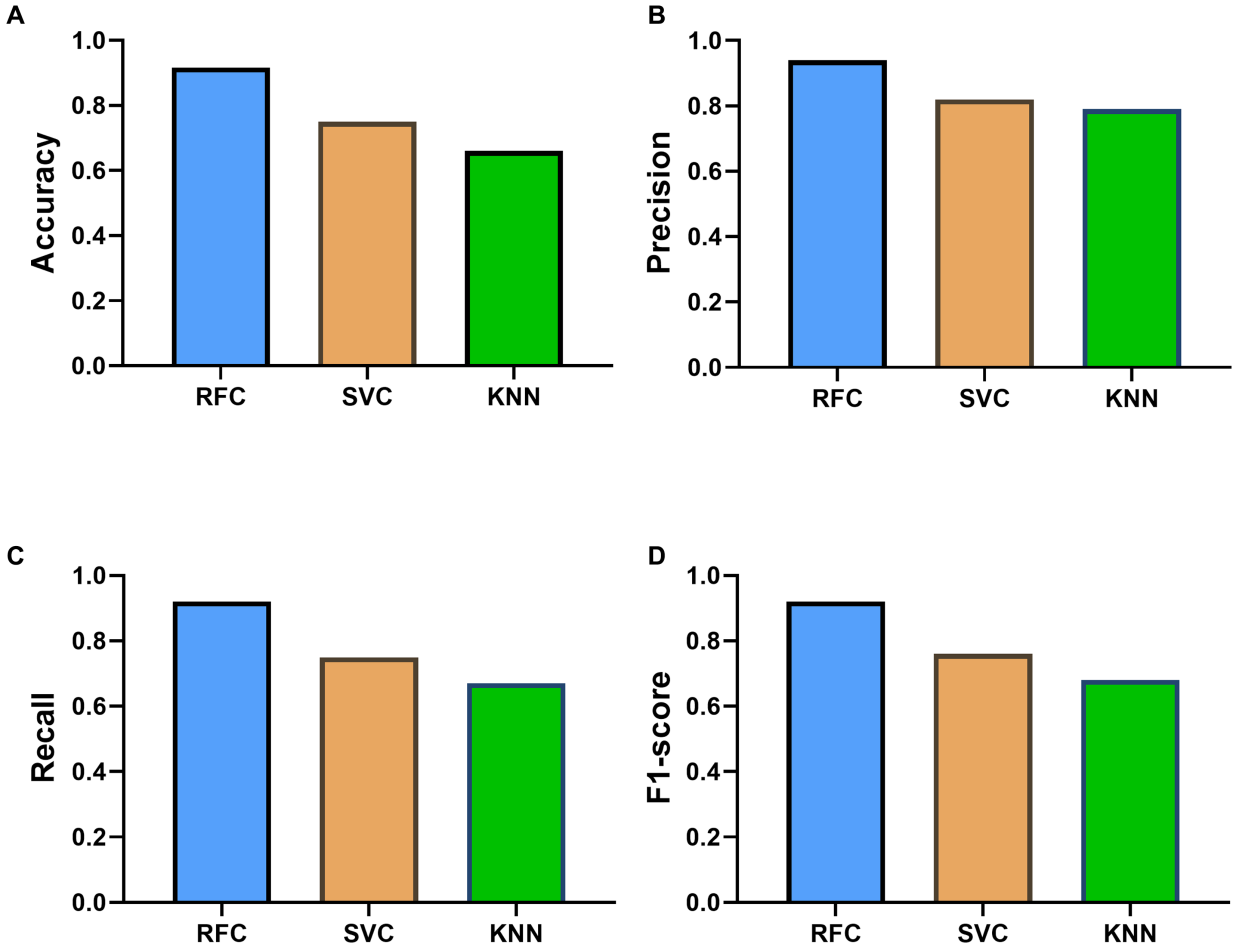
Performance Comparison of Different Machine Learning Models—Random Forest Classifier (RFC), Support Vector Classifier (SVC), and K-Nearest Neighbors (KNN)—in Predicting Insomnia Severity Levels based on the Constitution in Chinese Medicine (CCM) Scale Score. Panel **(A–D)** showed the results of accuracy, precision, recall and F1-score, respectively.

The comparative performance of various classifiers is presented in Figure 2. Among all the models evaluated in this study, the RFC outperformed others, yielding an accuracy score of 0.916. The superior performance of RFC was achieved with hyperparameters set at a maximum depth of None, a minimum sample split of 5, and 100 estimators. This accentuates the promising utility of RFC for classifying the severity of insomnia grounded on Traditional Chinese Medicine (TCM) constitution scores. In contrast, the SVC and KNN classifiers, despite being fine-tuned based on their optimal parameters, rendered relatively lower accuracies of 0.75 and 0.66, respectively.

**Figure 3:**
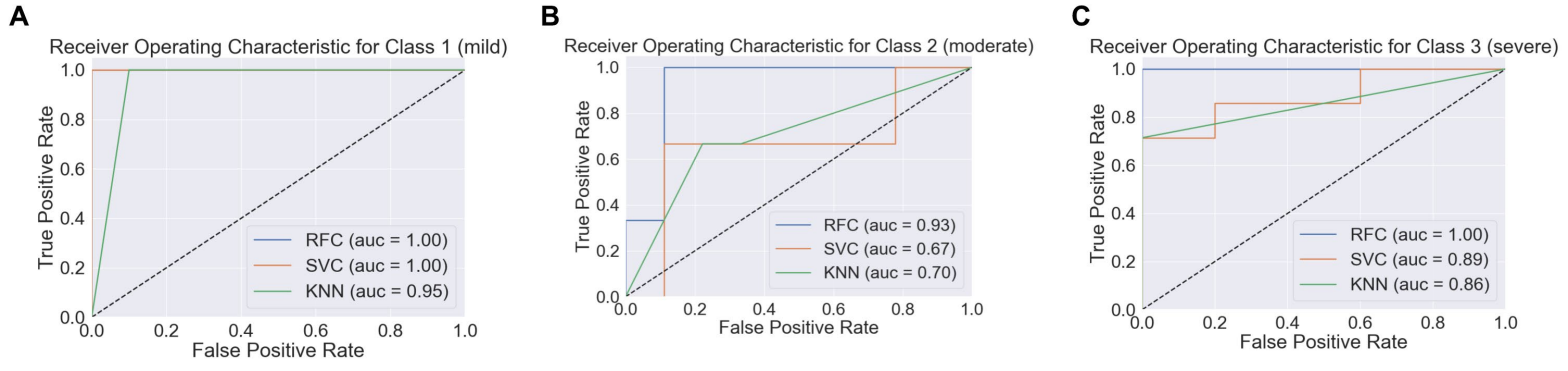
Receiver operating characteristic curves (ROC) for three machine learning models. Panel **(A–C)** represented the insomnia severity classification of class 1 (Mild Insomnia), class 2 (Moderate Insomnia), and Class 3 (Severe Insomnia) against other classes, with AUC values indicating the models’ discriminatory power, respectively.

Upon further evaluation, the RFC displayed superior weighted averages across precision, recall, and F1-score, suggesting its robust performance across all categories of insomnia severity. The SVC and KNN classifiers also demonstrated commendable performance, as indicated by their weighted averages; however, they were marginally outperformed by the RFC. More specifically, RFC presented a weighted average precision of 0.94, a recall of 0.92, and a F1-score of 0.92, underpinning its high predictability and low false positive rate. Furthermore, the predictive performance of the models was also evaluated using AUC-ROC. For differentiating mild insomnia from other classes, all three models demonstrated exemplary performance, with RFC and SVC achieving a perfect AUC of 1, and KNN closely following with an AUC of 0.95 (Figure 3A). When distinguishing moderate insomnia from the other severities, RFC still performed significantly well with an AUC of 0.93, whereas SVC and KNN yielded lower AUCs of 0.67 and 0.7, respectively (Figure 3B). In the classification of severe insomnia against the other classes, RFC achieved an AUC of 1, showcasing its superior predictive capability, while SVC and KNN showed commendable performance with AUCs of 0.89 and 0.86, respectively (Figure 3C). This denotes that, in this context, RFC tends to provide a more accurate and reliable classification prediction of insomnia severity.

**Figure 4:**
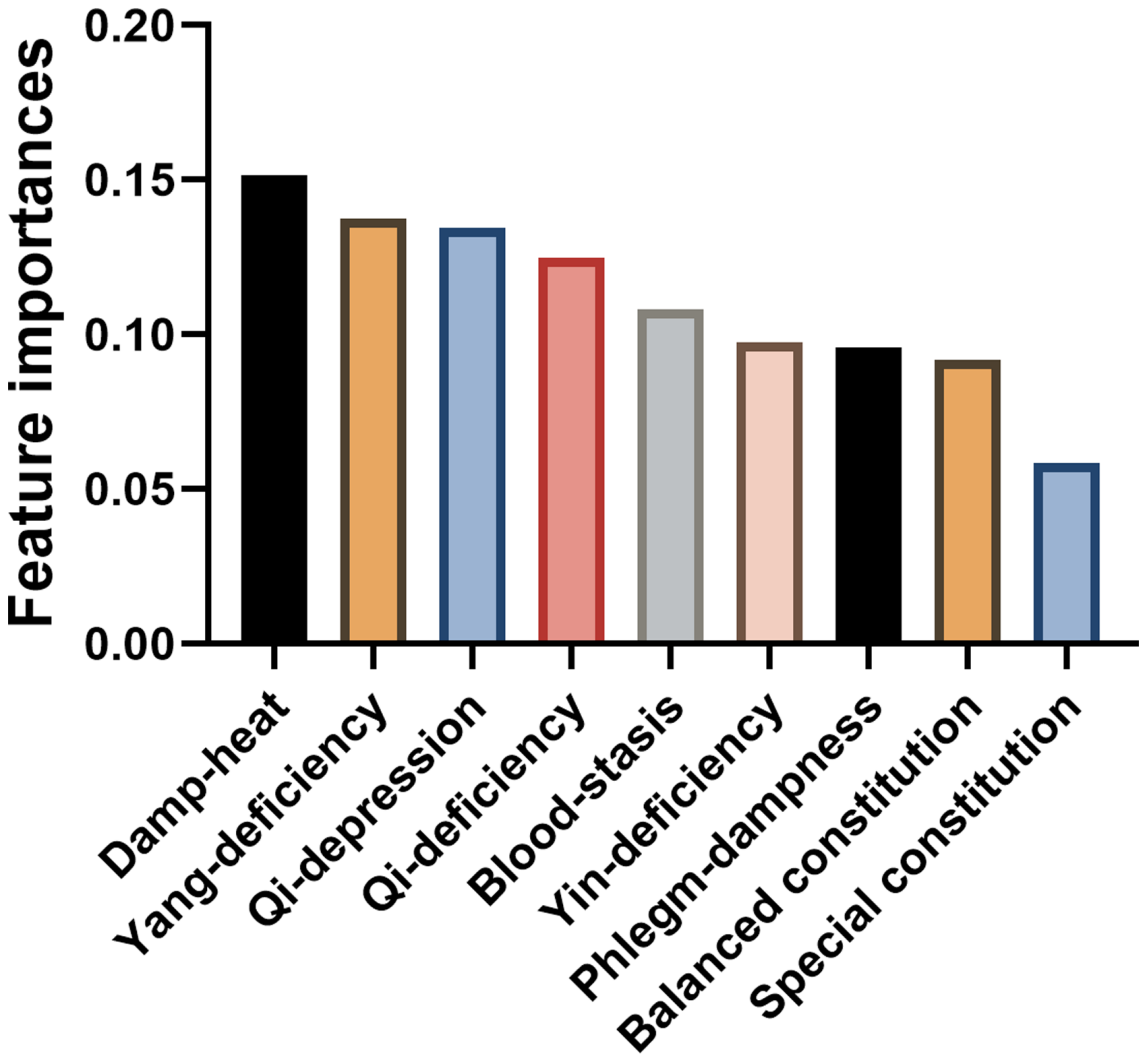
Feature importance values of traditional Chinese medicine (TCM) constitution classifications in random forest classifier (RFC).

The RFC model’s insightful exploration delineated a hierarchy of TCM constitution classifications based on their predictive potency and correlation with insomnia severity. Emphasizing this, the Damp-heat constitution manifested the highest feature importance, registering at 0.1514. This was closely followed by Yang-deficiency, Qi-depression, Qi-deficiency, and Blood-stasis constitutions, with importance values of 0.1374, 0.1346, 0.1249, and 0.1082 respectively, all surpassing the benchmark of 0.10. The substantial feature importance of these TCM constitution classifications, therefore, underscores their significant predictive role and potent correlation with insomnia severity (Figure 4).

## 4. Discussion

The use of machine learning models in the field of medical research and particularly in the realm of TCM represents a significant advance in contemporary medicine (27). In the current study, we used three established machine learning models: Random Forest, Support Vector Machine, and K-Nearest Neighbors, to predict the severity of insomnia based on TCM constitution scores. Among these, RFC emerged as the most predictive model, demonstrating superior accuracy compared to SVC and KNN. Random forest, a powerful ensemble machine learning model, has been widely used in various medical fields, including in the diagnosis and prognosis of diseases like cancer (28), cardiovascular disease (29), and diabetes (30). Its high performance can be attributed to its ability to handle high-dimensional data, capture non-linear relationships, and accommodate potential interactions among features (31). These attributes are particularly important when dealing with complex medical data, where a multitude of factors interplay to determine health outcomes (32). Additionally, random forest has the added advantage of providing feature importance, offering insight into which predictors most significantly impact the predicted outcome (33). Thus, random forest presents a potent tool for predicting insomnia severity in the context of TCM constitutions.

The importance of TCM constitution classifications in predicting insomnia severity cannot be understated. Our results showed that certain TCM constitution classifications such as ‘Qi-deficiency’, ‘Yang-deficiency’, ‘Damp-heat’, ‘Blood-stasis’, and ‘Qi-depression’ exhibited significant feature importance, each greater than 0.10. This suggests that these specific TCM classifications might play a key role in contributing to the severity of insomnia.

Interestingly, modern medical research supports this observation. For instance, a study published in 2015 established a link between Qi-deficiency, which relates to energy levels and fatigue in TCM, and increased severity of insomnia (34). Similarly, ‘Yang-deficiency’ which in TCM is associated with cold sensations and poor circulation, has been shown to affect sleep quality, particularly in the elderly population (35). ‘Damp-heat’, another TCM constitution classification, refers to a state of imbalance in the body often associated with inflammation (36). This imbalance has been linked to sleep disturbances in a study published in 2021 (37). Blood-stasis, representing a stagnation or slowing down of circulation in TCM, has been associated with sleep apnea in a recent study (38), which could lead to disrupted sleep and increased insomnia severity. Lastly, ‘Qi-depression’, a state of emotional stagnation in TCM, has been associated with psychiatric conditions such as depression and anxiety, which are well-known contributors to insomnia (39, 40).

The application of this prediction model, particularly in clinical treatment and prevention of insomnia, could be wide-reaching. As we have demonstrated, the model can effectively predict insomnia severity based on TCM constitution classifications. These insights could guide clinicians in tailoring individual treatment strategies for patients suffering from insomnia, taking into consideration the identified important TCM constitution classifications. Moreover, it could assist in patient stratification, helping healthcare professionals to identify individuals at higher risk of severe insomnia and therefore needing more immediate or intensive interventions (41). For example, for patients showing signs of ‘Qi-deficiency’ and ‘Yang-deficiency’, treatment strategies could focus on addressing the corresponding imbalances, using modalities such as herbal remedies, acupuncture, or lifestyle adjustments known to help correct these specific deficiencies. Similarly, for those showing ‘Damp-heat’, ‘Blood-stasis’, and ‘Qi-depression’ classifications, targeted therapies could be implemented to address these conditions, which in turn, could ameliorate the severity of insomnia (42).

Importantly, the use of such a model can also guide preventative measures (43, 44). By identifying the at-risk population, preventive interventions can be implemented early, before the onset of severe insomnia. Such proactive management could potentially reduce the burden of insomnia on both the individual and healthcare system.

Furthermore, while our model has been applied to insomnia in the context of TCM, the same methodology can be applied to other health conditions where TCM constitution classifications play a role. This opens the door to a range of potential applications, further enhancing the utility of TCM constitution classifications in modern healthcare.

Nevertheless, further validation of the model in different populations and clinical settings is necessary to ascertain its generalizability. As we continue to integrate traditional and modern medical knowledge, models such as the one presented in this study will be instrumental in enabling a more nuanced understanding of health and disease, ultimately benefiting patient care.

## 5. Conclusion

In conclusion, this study illuminates the potential of employing machine learning models, particularly the Random Forest, alongside TCM constitution classifications to enhance the management of insomnia. The substantial predictive capacity of TCM constitution types such as Damp-heat and Yang-deficiency suggests a pathway towards more personalized, and therefore potentially more effective, treatment approaches. These predictive models could serve as valuable tools in both the clinical decision-making process and the formulation of targeted preventative measures. While the results are encouraging, further validation in diverse patient populations remains essential to ensure their robust applicability.

## Data availability statement

The raw data supporting the conclusions of this article will be made available by the authors, without undue reservation.

## Ethics statement

The studies involving humans were approved by Ethics Committee of the Shanghai Minhang District Integrated Traditional Chinese and Western Medicine Hospital (Ethics Reference No. 2021-007). The studies were conducted in accordance with the local legislation and institutional requirements. The participants provided their written informed consent to participate in this study.

## Author contributions

SL: Data curation, Funding acquisition, Investigation, Writing – original draft. PZ: Data curation, Investigation, Writing – review & editing. GC: Data curation, Investigation, Writing – review & editing. JL: Data curation, Investigation, Writing – review & editing. TH: Conceptualization, Writing – review & editing. WT: Conceptualization, Writing – review & editing.

## References

[1] Sutton EL. Insomnia. Med Clin North Am. (2014) 98:565–81. doi: 10.1016/j.mcna.2014.01.008 24758961

[2] Riemann D Benz F Dressle RJ Espie CA Johann AF Blanken TF. Insomnia disorder: state of the science and challenges for the future. J Sleep Res. (2022) 31:e13604. doi: 10.1111/jsr.13604, PMID: 35460140

[3] Schubert CR Cruickshanks KJ Dalton DS Klein BE Klein R Nondahl DM. Prevalence of sleep problems and quality of life in an older population. Sleep. (2002) 25:889–93. doi: 10.1093/sleep/25.8.48 12489896

[4] Boland E Goldschmied J Kayser MS Gehrman PR. Precision medicine for insomnia. Sleep Med Clin. (2019) 14:291–9. doi: 10.1016/j.jsmc.2019.04.001 31375199

[5] Asarnow LD Manber R. Cognitive behavioral therapy for insomnia in depression. Sleep Med Clin. (2019) 14:177–84. doi: 10.1016/j.jsmc.2019.01.009, PMID: 31029185 PMC6487874

[6] Krystal AD. Optimizing treatment for insomnia. J Clin Psychiatry. (2021) 82:EI20008BR4C. doi: 10.4088/JCP.EI20008BR4C 34255942

[7] Ni X Shergis JL Zhang AL Guo X Lu C Li Y. Traditional use of Chinese herbal medicine for insomnia and priorities setting of future clinical research. J Altern Complement Med. (2019) 25:8–15. doi: 10.1089/acm.2018.0249 30376350

[8] He W Li M Zuo L Wang M Jiang L Shan H. Acupuncture for treatment of insomnia: an overview of systematic reviews. Complement Ther Med. (2019) 42:407–16. doi: 10.1016/j.ctim.2018.12.020, PMID: 30670275

[9] Bai MH Wong W Hou SJ Zheng YF Li QR Li ZQ. Development and evaluation of short form of constitution in Chinese medicine questionnaire: a national epidemiological survey data of 21 948 case. J Tradit Chin Med. (2022) 42:122–31. doi: 10.19852/j.cnki.jtcm.20211228.001, PMID: 35294132 PMC10164641

[10] Sun Y Zhao Y Xue SA Chen J. The theory development of traditional Chinese medicine constitution: a review. J. Trad. Chin. Med. Sci. (2018) 5:16–28. doi: 10.1016/j.jtcms.2018.02.007

[11] Wong W Lam CLK Wong VT Yang ZM Ziea ET Kwan AKL. Validation of the constitution in Chinese medicine questionnaire: does the traditional Chinese medicine concept of body constitution exist? Evid Based Complement Alternat Med. (2013) 2013:481491. doi: 10.1155/2013/481491 23710222 PMC3655622

[12] Li L Yao H Wang J Li Y Wang Q. The role of Chinese medicine in health maintenance and disease prevention: application of constitution theory. Am J Chin Med. (2019) 47:495–506. doi: 10.1142/S0192415X19500253, PMID: 31023059

[13] Singh A Zhao K. Treatment of insomnia with traditional Chinese herbal medicine. Int Rev Neurobiol. (2017) 135:97–115. doi: 10.1016/bs.irn.2017.02.006 28807167

[14] Zhang CH Chun-Guang YU Pei-Yao LI Ling WANG Hui-Xin DING Wen-Wen ZHAO. Research status of traditional Chinese medicine constitution theory in insomnia. Journal of integrative. Nursing. (2019) 1:51–8. doi: 10.35437/intnur.issn.2663-4481.2019.01.01.07

[15] Poon MMK Chung KF Yeung WF Yau VHK Zhang SP. Classification of insomnia using the traditional chinese medicine system: a systematic review. Evid Based Complement Alternat Med. (2012) 2012:735078. doi: 10.1155/2012/735078 22899958 PMC3414091

[16] He Q Yang YF Wu CL. A clinical trial of treatment of primary insomnia of patients with qi-stagnation constitution by shallow acupuncture combined with ear-acupoint pellet-pressing. Zhen Ci Yan Jiu. (2019) 44:293–6. doi: 10.13702/j.1000-0607.170614 31056884

[17] Liu C Qu J Chen L Liu R. Analysis of sleep quality and TCM constitution characteristics in 258 outpatients: a cross-sectional study based on outpatient cases. Appl Bionics Biomech. (2022) 2022:2952531. doi: 10.1155/2022/2952531 35989714 PMC9385366

[18] Ferdous M. Debnath J. Chakraborty N.R. Machine learning algorithms in healthcare: a literature survey. In 2020 11th international conference on computing, communication and networking technologies (ICCCNT). (2020).

[19] Kaur P Kumar R Kumar M. A healthcare monitoring system using random forest and internet of things (IoT). Multimed Tools Appl. (2019) 78:19905–16. doi: 10.1007/s11042-019-7327-8

[20] Razzaghi T Roderick O Safro I Marko N. Multilevel weighted support vector machine for classification on healthcare data with missing values. PLoS One. (2016) 11:e0155119. doi: 10.1371/journal.pone.0155119, PMID: 27195952 PMC4873242

[21] Tayeb S. Pirouz M. Sun J. Hall K. Chang A. Li J.. Toward predicting medical conditions using k-nearest neighbors. In 2017 IEEE international conference on big data (big data) (2017).

[22] Khateeb N. Usman M., Efficient heart disease prediction system using K-nearest neighbor classification technique, in Proceedings of the international conference on big data and internet of thing. (2017), Association for Computing Machinery: London, United Kingdom. p. 21–26.

[23] Yang L Wu H Jin X Zheng P Hu S Xu X. Study of cardiovascular disease prediction model based on random forest in eastern China. Sci Rep. (2020) 10:5245. doi: 10.1038/s41598-020-62133-5, PMID: 32251324 PMC7090086

[24] Xu F Zhang Y Cui W Yi T Tang Z Dong J. The association between metabolic syndrome and body constitution in traditional Chinese medicine. Eur J Integr Med. (2017) 14:32–6. doi: 10.1016/j.eujim.2017.08.008

[25] Klimm HD Dreyfus JF Delmotte M. Zopiclone versus nitrazepam: a double-blind comparative study of efficacy and tolerance in elderly patients with chronic insomnia. Sleep. (1987) 10:73–8. doi: 10.1093/sleep/10.suppl_1.73, PMID: 3326118

[26] Hausswirth C Nesi X Dubois A Duforez F Rougier Y Slattery K. Four weeks of a neuro-meditation program improves sleep quality and reduces hypertension in nursing staff during the COVID-19 pandemic: a parallel randomized controlled trial. Front Psychol. (2022) 13:854474. doi: 10.3389/fpsyg.2022.854474, PMID: 35645851 PMC9130829

[27] Chu X Sun B Huang Q Peng S Zhou Y Zhang Y. Quantitative knowledge presentation models of traditional Chinese medicine (TCM): a review. Artif Intell Med. (2020) 103:101810. doi: 10.1016/j.artmed.2020.101810, PMID: 32143806

[28] Murugan A Nair SAH Kumar KPS. Detection of skin Cancer using SVM, random Forest and kNN classifiers. J Med Syst. (2019) 43:269. doi: 10.1007/s10916-019-1400-8 31273532

[29] Su X Xu Y Tan Z Wang X Yang P Su Y. Prediction for cardiovascular diseases based on laboratory data: an analysis of random forest model. J Clin Lab Anal. (2020) 34:e23421. doi: 10.1002/jcla.23421 32725839 PMC7521325

[30] VijiyaKumar K.. Random Forest algorithm for the prediction of diabetes. In 2019 IEEE international conference on system, computation, automation and networking (ICSCAN). 2019.

[31] Speiser JL Miller ME Tooze J Ip E. A comparison of random Forest variable selection methods for classification prediction modeling. Expert Syst Appl. (2019) 134:93–101. doi: 10.1016/j.eswa.2019.05.028, PMID: 32968335 PMC7508310

[32] Parmar A. Katariya R. Patel V. A review on random Forest: an ensemble classifier. In International conference on intelligent data communication technologies and internet of things (ICICI) 2018. (2019). Cham: Springer International Publishing.

[33] Alam MZ Rahman MS Rahman MS. A random Forest based predictor for medical data classification using feature ranking. Inform Med Unlocked. (2019) 15:100180. doi: 10.1016/j.imu.2019.100180

[34] Ye Q Zhou J Yuan X Yuan C Yang X. Efficacy of Zhenjingdingzhi decoction in treating insomnia with qi-deficiency of heart and gallbladder: a randomized, double-blind, controlled trial. J Tradit Chin Med. (2015) 35:381–8. doi: 10.1016/s0254-6272(15)30113-8, PMID: 26427106

[35] Yingshuai LI Yan LI. Progress in the study of -deficiency constitution in terms of traditional Chinese medicine: a narrative review. J Tradit Chin Med. (2023) 43:409–16. doi: 10.19852/j.cnki.jtcm.20221206.001, PMID: 36994531 PMC10012185

[36] Zhao H Zong Y Li W Wang Y Zhao W Meng X. Damp-heat constitution influences gut microbiota and urine metabolism of Chinese infants. Heliyon. (2023) 9:e12424. doi: 10.1016/j.heliyon.2022.e12424, PMID: 36755610 PMC9900481

[37] Chang Y Shen W. Clinical observation of acupuncture combined with Longdan Xiegan decoction in the treatment of insomnia of hepatobiliary damp-heat type. Minerva Surg. (2021) 1–3. doi: 10.23736/S2724-5691.21.09238-8, PMID: 34790938

[38] Li-na K Na-na H Ya-jun C Xi-jun H Min F Zhao S. Acupuncture plus bloodletting therapy for insomnia in blood stasis constitution: a clinical study. J Acupunct Tuina Sci. (2018) 16:38–42. doi: 10.1007/s11726-018-1021-7

[39] Kondo T Tokunaga S Sugahara H Yoshimasu K Kanemitsu Y Kubo C. Identification of visceral patterns in patients with stress-related disorders. Integr Med Int. (2015) 1:185–98. doi: 10.1159/000375532

[40] Liu Z Cao L Wu J He Y Tu J Huang J. Association of qi-stagnation constitution and subjective sleep characteristics with mild cognitive impairment among elderly in community: a cross-sectional study. Eur J Integr Med. (2023) 59:102232. doi: 10.1016/j.eujim.2023.102232

[41] Wang Q Bai M Yang Y Liang X Sun P Han J. Application of TCM constitution in lifetime health maintenance. J Trad Chin Med Sci. (2018) 5:6–15. doi: 10.1016/j.jtcms.2018.02.006

[42] Sang X Wang Z Liu S Wang R. Relationship between traditional Chinese medicine(TCM)constitution and TCM syndrome in the diagnosis and treatment of chronic diseases. Chin Med Sci J. (2018) 33:114–9. doi: 10.24920/21806 29976281

[43] Wang Q. Individualized medicine, health medicine, and constitutional theory in Chinese medicine. Front Med. (2012) 6:1–7. doi: 10.1007/s11684-012-0173-y, PMID: 22460443

[44] Li L Wang Z Wang J Zheng Y Li Y Wang Q. Enlightenment about using TCM constitutions for individualized medicine and construction of Chinese-style precision medicine: research progress with TCM constitutions. Sci China Life Sci. (2021) 64:2092–9. doi: 10.1007/s11427-020-1872-7, PMID: 33400060

